# Plasma neuronal specific enolase: a potential stage diagnostic marker in human African trypanosomiasis

**DOI:** 10.1093/trstmh/tru065

**Published:** 2014-04-30

**Authors:** Jeremy M. Sternberg, Julia A. Mitchell

**Affiliations:** Institute of Biological and Environmental Sciences, University Of Aberdeen, Aberdeen AB24 2TZ, UK

**Keywords:** African trypanosomiasis, Neuronal specific enolase, Stage

## Abstract

**Background:**

This study was carried out to determine the potential of neuronal specific enolase (NSE) as a stage diagnostic marker in human African trypanosomiasis.

**Methods:**

Plasma and cerebrospinal fluid were obtained from a cohort of *Trypanosoma brucei rhodesiense*-infected patients and non-infected controls. Neuronal specific enolase concentrations were measured by ELISA and analysed in relation to diagnosis and disease-stage data.

**Results:**

Plasma NSE concentration was significantly increased in late-stage patients (median 21 ng/ml), compared to the control (median 11 ng/ml), but not in early-stage patients (median 5.3 ng/ml). Cerebrospinal fluid NSE concentration did not vary between stages.

**Conclusion:**

Plasma NSE is a potential stage diagnostic in this cohort and merits further investigation.

## Introduction

Human African trypanosomiasis (HAT) is caused by African trypanosomes of the sub-species *Trypanosoma brucei rhodesiense* and *Trypanosoma brucei gambiense*. In both sub-species, infections progress through two stages, the early or haemolymphatic stage and the late or meningoencephalitic stage where parasites invade the central nervous system (CNS).^1^ In both clinical cases and experimental models the late stage of disease is associated with neuro-inflammatory pathology, including astrogliosis and neuronal degeneration.^2–4^ The detection of CNS invasion by trypanosomes is of enormous clinical diagnostic importance, as chemotherapy for late-stage HAT carries increased toxicity risks than that for early-stage. In *T.b. rhodesiense* infections, late-stage disease is treated with the arsenical melarsoprol, which has a drug-induced fatality rate that may be as high as 5%.^5^ In *T.b. gambiense* infection, the introduction of the nifurtimox-eflornothine combination treatment has reduced toxic side-effects, but there remains a significant logistic challenge to making this treatment available on a large scale.^6^ Therefore, prompt and correct staging of diagnosed HAT cases is of vital clinical importance.

Currently, staging depends entirely on the analysis of cerebrospinal fluid (CSF), and all patients with a positive HAT diagnosis undergo lumbar puncture. The current late-stage diagnostic criteria are the presence of trypanosomes in the CSF or a CSF leucocytosis of greater than 5 cells/µl.^7^ Various other CSF markers of neuroinflammation have also been shown to have stage diagnostic potential, either singly^6^ or in multi-analyte panel assays.^8^ However, these methods all continue to rely on the invasive procedure of lumbar puncture to obtain CSF. Given that the initial diagnosis of HAT is made using a blood sample, it would ideal to also obtain disease-stage data from serum or plasma.^9^

In this study, the CSF and plasma concentrations of the enzyme neuronal specific enolase (NSE) were studied retrospectively in patients infected with *T.b. rhodesiense*. Neuronal specific enolase is an isoenzyme (γγ isoform) of enolase that is found in high concentrations in neurons and neuroendocrine cells, and is a sensitive marker for neuronal damage.^10^ It is able to diffuse rapidly across the blood-brain barrier,^11^ and would therefore be expected to be a sensitive plasma marker for CNS trauma. Plasma NSE has also been shown to be a useful marker for neuronal injury both in clinical cases^11^ and in experimental models.^13^ There have been no previous studies of this marker in HAT infections.

## Methods

The 143 plasma and CSF samples used in this study were obtained from the LIRI Clinic, Tororo and Serere Health Centre in eastern Uganda. These samples were from a larger epidemiological study and recruitment and the associated clinical and pathophysiological data collection have been described elsewhere.^14^ Thirty-seven control plasma samples were obtained from patients at the clinic who were suspected of having HAT, but later diagnosed as non-infected. Diagnosis of HAT was made by microscopic detection of trypanosomes. Stage was determined using the WHO criteria in which patients with trypanosomes in the CSF and/or a cell count of >5 cells/mm^3^ were classified as late- stage.^15^ Early-stage infection was treated with suramin and late-stage infection with melarsoprol (as described in MacLean et al.^16^), with post-treatment samples obtained at 36–42 days post-admission. Individual written informed consent was obtained from all study participants. Individuals with malarial parasitaemia and microfilaraemia were excluded from the study.

Blood samples obtained before treatment commenced were collected into K-EDTA Vacutainers (Vacuette, Greiner, Stroud, UK) and centrifuged for 10 min at 3000 g. Platelet-depleted plasma were aliquoted and frozen within 1 h of collection in liquid nitrogen. Cerebrospinal fluid samples, obtained as part of normal stage diagnosis, were also frozen and stored in liquid nitrogen. All samples were kept in liquid nitrogen for the duration of the field study (6–18 months), after which they were shipped to the UK, thawed once, aliquoted and stored at −80**°**C until analysis.

Neuronal specific enolase concentrations were analysed using a sandwich ELISA specific for the γ subunit (Kit 0050, Alpha Diagnostics, San Antonio, TX, USA) according to the manufacturer's instructions. Samples were analysed in triplicate aliquots of 25 µl. Concentrations of NSE below the detection limit of the assay were scored as 0.5 x limit of detection for analysis.^17^ Haemoglobin concentrations in plasma samples were measured in triplicate using the alkaline hematin detergent method.^18^ Data analysis was carried out using JMP10.0 (SAS Institute Inc., Cary, NC, USA).

## Results and Discussion

The study analysed plasma and CSF from 143 *T.b. rhodesiense* HAT-patients and 37 non-infected controls. Of the 143 HAT patients, 109 cases were diagnosed as late-stage and 34 as early-stage. The demographic details of the patient group are provided in the Supplementary data. There were no recorded relapses over a one-year follow-up period, indicating the accuracy of the early-stage diagnosis.

**Figure 1.**
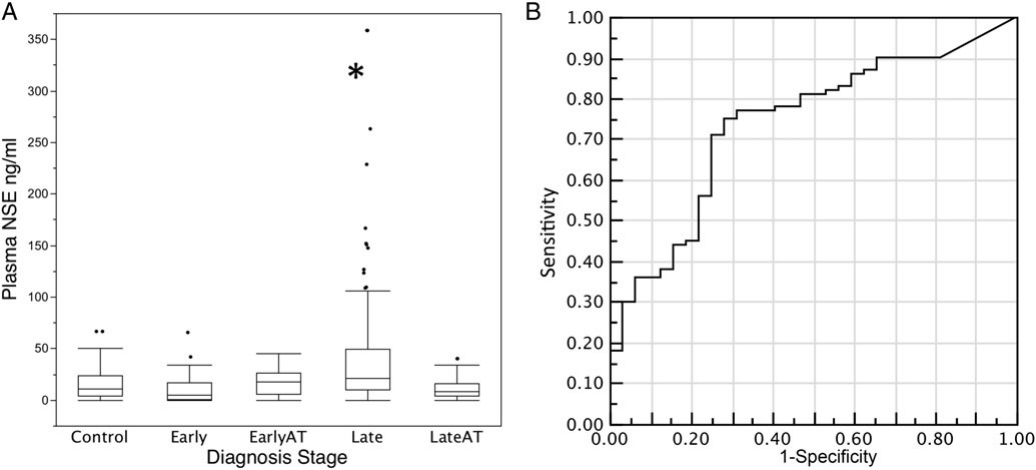
Neuronal specific enolase (NSE) concentrations in *Trypanosoma brucei rhodesiense* HAT patients on admission and after a chemotherapeutic treatment (AT).

Patients in the late-stage showed significantly elevated levels of plasma NSE (median 21 ng/ml) compared to patients in the early-stage (median 5.3 ng/ml; p<0.001; Mann-Whitney test) and control individuals (median 11.0 ng/ml; p<0.01) (Figure 1A). No relationship was detected between plasma NSE concentration and patient age or gender (standard least squares model), nor was any relationship detected with recorded thick-film parasitaemia data (Spearmann ρ −0.07). Control plasma NSE concentrations were consistent with previous reports.^12,19^ The increased concentration levels of plasma NSE in late-stage cases was similar to levels detected in patients with traumatic brain injury^10^ or acute ischaemic stroke.^12^ As haemolytic plasma may contain red blood cell γα isoforms of enolase,^20^ plasma haemoglobin concentrations were determined. Haemoglobin levels in the study samples were low (median 6 mg/dl; IQR 5–9 mg/dl) and showed no relationship with the detected NSE concentrations (Spearmann ρ −0.12).

(A) Plasma NSE concentration in control (n=37), early (n=34) and late-stage (n=109) are shown*.* Boxes are median and IQR, whiskers 10th and 90th percentiles and dots represent outliers. * indicates a significant increase over the control (p<0.01). (B) Receiver operating characteristic (ROC) plot of sensitivity and specificity of plasma NSE concentration as a diagnostic for late-stage HAT.

Neuronal specific enolase concentrations were also measured in plasma prepared from patient blood samples at the time of discharge from treatment (36–42 days post-admission). In these samples, there was no longer any significant difference between early-stage (median 6.1 ng/ml) and late-stage (median 8.2 ng/ml) NSE concentrations, which were also not significantly different to control concentrations (11 ng/ml). When plasma NSE concentrations of individual patients were compared, for early-stage patients there were no significant change in plasma NSE concentration after treatment, whereas there was a significant reduction in NSE concentration in the late-stage patients (Wilcoxon Signed Rank test; p<0.001).

Plasma NSE was predictive of disease stage (OR per unit increase in NSE was 1.04 [95% CI 1.02–1.08; p<0.0001]), but the large number of outliers in both stages resulted in limited diagnostic potential. The area under the receiver operator curve (ROC) was 0.73 (Figure 1B), and at the optimal diagnostic cut-off value for late-stage of 10.4 ng/ml, NSE sensitivity was 75% and specificity was 72%. While this is not adequately discriminating to be used in a clinical diagnosis, it does represent the first demonstration of a late-stage specific marker in blood.

While the plasma NSE concentrations showed a stage-specific increase in HAT patients, in the CSF samples no effect was observed. Cerebrospinal fluid concentrations of NSE were mostly low (early-stage 1.2 ng/ml [IQR 1.2–1.2 ng/ml] and late-stage 1.2 ng/ml [IQR 1.2–7.6 ng/ml]) and consistent with previously reported control values.^21,22^ Interestingly, high levels NSE have been reported after traumatic brain injury (>100 ng/ml) in ventricular CSF, but no data is available for lumbar CSF. In neurological diseases, NSE concentrations in lumbar CSF are often low (<10 ng/ml).^23^ Given the low levels of NSE detected in the CSF, it is possible that the source of NSE in the plasma samples was outside the CNS. Although peripheral neuronal degeneration has not been investigated in HAT, there is good evidence for CNS neuronal degeneration in experimental trypanosomiasis.^24^ This requires further investigation in experimental models of infection.

### Conclusion

In *T.b. rhodesiense* HAT patients, plasma NSE levels are significantly increased compared to the control in late-stage, but not early-stage patients. As such plasma NSE represents a potential stage diagnostic, particularly if complementary markers can be identified to increase its diagnostic power. Furthermore, the normalisation of plasma NSE after treatment of late-stage cases merits further investigation as a potential treatment efficacy marker. Model infection studies are now required to identify the source of NSE and pathophysiological mechanisms underlying its release.

## Supplementary data

Supplementary data are available at *Transactions Online* (http://trstmh.oxfordjournals.org/).

Supplementary Data
