# Supplementary material for: Plasma neuronal specific enolase: a potential stage diagnostic marker in human African trypanosomiasis
Source: Trans R Soc Trop Med Hyg. 2014 Apr 30;108(7):449–52. doi: 10.1093/trstmh/tru065 (PMC4342681; doi:10.1093/trstmh/tru065)
Supplement: Supplementary Data [file supp_108_7_449__index.html]

Plasma neuronal specific enolase: a potential stage diagnostic marker in human African trypanosomiasis — Plasma neuronal specific enolase: a potential stage diagnostic marker in human African trypanosomiasis — Supplementary Data 

# Plasma neuronal specific enolase: a potential stage diagnostic marker in human African trypanosomiasis

## Supplementary Data

Supplementary Data

**Files in this Data Supplement:**

- Supplementary Data - Docx file
