# Supplementary material for: Plasma neuronal specific enolase: a potential stage diagnostic marker in human African trypanosomiasis
Source: Trans R Soc Trop Med Hyg. 2014 Apr 30;108(7):449–52. doi: 10.1093/trstmh/tru065 (PMC4342681; doi:10.1093/trstmh/tru065)
Supplement: Supplementary Data [file supp_tru065_tru065supp.docx]

Supplementary Data

Demographic characteristics of HAT patients

Early Stage Late Stage

Number 34 109

Male:female 14:20 49:60

Age (Median IQR) 20(13-43) 23(13-40)
